# A genome-wide mutation analysis method enabling high-throughput identification of chemical mutagen signatures

**DOI:** 10.1038/s41598-018-27755-w

**Published:** 2018-06-25

**Authors:** Shoji Matsumura, Yurika Fujita, Masayuki Yamane, Osamu Morita, Hiroshi Honda

**Affiliations:** 0000 0001 0816 944X grid.419719.3 R&D, Safety Science Research, Kao Corporation, 2606 Akabane, Ichikai-Machi, Haga-Gun, Tochigi, 321-3497 Japan

## Abstract

Trinucleotide mutational signatures extracted from cancer genomes provide clues useful in understanding the roles of mutagens and mutagenic mechanisms in cancer development. The lack of a simple method for genome-wide analysis of alterations induced by mutagens hampers the identification of trinucleotide signatures of mutagen exposure and evaluation of their relationships with human cancers. Here, we describe a novel approach to facilitate analysis of chemically induced mutations in bacterial cells by detection of increased frequencies of base substitutions after mutagen exposure, using paired-end overlapping next-generation sequencing. DNA samples from *Salmonella typhimurium* strain TA100, exposed to three alkylating agents, ethylnitrosourea (ENU), methylnitrosourea (MNU), and ethyl methansulphonate (EMS), were analysed. The G:C > A:T mutation frequency was increased in all samples, whereas A:T base pair substitution frequencies were increased specifically in samples exposed to ENU, consistent with previous reports. Mutation patterns in the context of 96 possible trinucleotide formats in these samples exhibited a sharp peak corresponding to an NpCpY consensus sequence, which is similar to the mutational signature of alkylating agents in human cancer. These results indicate that our approach can be useful in facilitating the understanding of mechanisms underlying chemical mutagenicity and for identification of unknown causal mutagens in human cancer.

## Introduction

Cancer genomics studies have uncovered somatic mutations in cancer genomes and revealed their roles in cancer development^1^. Recent advances in next-generation sequencing (NGS) technology have enabled thorough characterisation of gene mutations in human cancer genomes^2–4^. These catalogues of mutations contain mixtures of the signatures generated by all mutational processes exerted on cancer genomes^5,6^. Alexandrov *et al*. developed a mathematical algorithm to separate and quantify each mutational signature from these catalogues^7^. Because the flanking sequence context of mutated bases affects their mutation rate, in these analyses, the bases immediately 5′ and 3′ of mutated nucleotides were also analysed, providing signatures of 96 distinct trinucleotide mutation formats (six types of substitution multiplied by four types of 5′ nucleotide and four types of 3′ nucleotide = 96). Patterns identified in this way are useful clues directly linking mutagens and cancers. In addition, extension of the concept of trinucleotide signatures to the evaluation of chemical mutagenicity would enable the classification of mutagens based on their mechanisms of action to be refined, leading to improved chemical safety assessment and regulation of mutagenic substances. By accumulating data on a variety of mutagens and cancers based on this concept, there is potential to systematically enrich understanding of the mechanisms underlying mutagen activity and their relationships with cancers, which could lead to refined cancer prediction and prevention.

Although retrospective extraction of mutation signatures from human cancer genomes has progressed, prospective identification of signatures caused by mutagen exposures has not advanced to the same extent^7^; therefore, there is a need for extraction of mutational signatures based on data from controlled experimental exposures. To date, mutational signatures of mutagen exposure have primarily been identified through whole-genome sequencing of cloned mutated cells; for example, primary cell cultures clonally expanded by 3T3 passaging^8^, and an animal model of carcinogenesis^9^. Although these methods allow identification of the mutagens causing signatures in specific cancers, they have limitations, particularly in their application to the evaluation of mutagenic chemical substances. First, the obtained signatures may have been influenced by the cell selection process and therefore not be representative of the exact mutation pattern caused by the relevant mutagen. Second, there are limitations in experimental throughput capacity when the techniques are applied to analyses of the effects of a variety of chemicals. Therefore, it is important to develop methodology to facilitate analysis of initial mutation patterns after exposure to mutagens. Development of such a method would be useful both for estimation of which mutagens cause different cancers and as a high-throughput mutagenicity test to identify the mechanisms of action of mutagens.

Conventionally, mutations induced by exposure to mutagens have primarily been evaluated by short term carcinogenicity studies, more specifically the bacterial Ames test system^10^. Such tests provide mutational information based on six mutation types (C > A, C > G, C > T, T > A, T > C, and T > G) and are useful for predicting mutagenicity in humans; however, they could not identify sufficient mutations to achieve high-resolution spectrum analyses, or to clarify trinucleotide signatures. This is because these tests depend on surrogate marker genes for mutation detection, which limits sequence context diversity and mutation quantity; therefore, the application of NGS to high-resolution analyses of chemically induced mutations on the whole-genome scale is of particular interest^11^. In this context, the most substantial challenge is the detection of chemically induced rare somatic mutations within the multitude of sequencing errors generated by NGS technology. A few studies have detected chemically induced mutations by whole-genome sequencing of isolated single cells^12^. Mimaki *et al*. analysed the trinucleotide mutational signatures induced by 1,2-dichloropropane using this approach, and identified similar patterns to a mutational signature in human cancer^13^. This study also demonstrated the utility of bacterial experiments for analysis of trinucleotide signatures that could be extrapolated to the human genome; however, as in the analyses using mammalian cells discussed above, these approaches require sampling and sequencing of multiple single cells to obtain mutational signatures at sufficient resolution. These approaches are expensive, particularly when applied to analyses of a number of mutagens; therefore, rather than isolating single cells, an alternative approach could be the identification of mutations based on information of single reads or read pairs derived from each DNA molecule. This could potentially enable detection of numerous mutations with minimal sampling and sequencing, and allow the adoption of low-error sequencing techniques, such as paired-end read stitching or duplex sequencing, to improve sequencing accuracy^14,15^.

Thus, in the present study, we developed a novel method that identifies the mutations from single reads to facilitate identification of mutation spectra generated by chemical exposures in a bacterial strain. Because the spontaneous mutation frequency is very low in bacteria^12,16^, it can be difficult to comprehensively discriminate true mutations from sequencing errors using this approach; therefore, we propose a method that focuses on the detection of an increase in mutation frequencies within the whole genome sequence compared with control samples, rather than accurate identification of each mutation. To increase sequencing accuracy, we adopted the widely used paired-end overlapping technique. We analysed the mutation patterns induced by alkylating agents as representative mutagens. We confirm the similarity of the resulting mutation patterns with those observed in historical bacterial studies and human cancer, and assess the applicability of our method for mutagenicity testing and the potential for extrapolation of the generated results to humans.

## Results

### Mutation detection workflow

**Figure 1:**
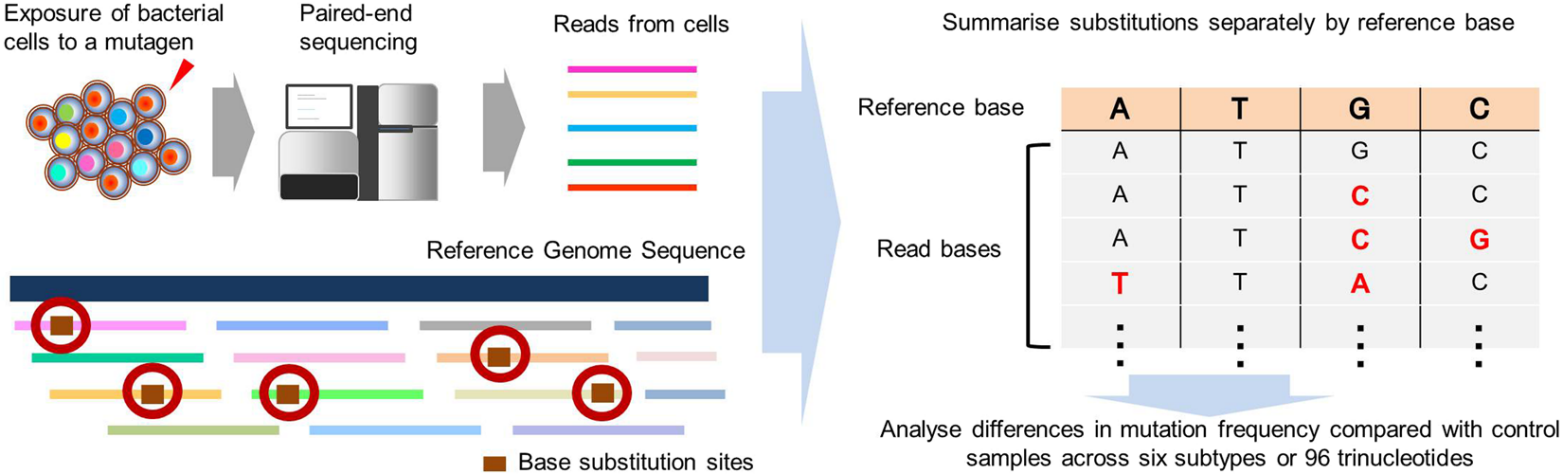
Overview of the mutation detection work flow. Genomic DNA samples were extracted from cell populations after chemical exposures and used for library construction and sequencing. Obtained reads were mapped to reference genome sequences, and base substitution sites were detected from each read. The ratio of each type of base substitution per 10^6^ bp was calculated by integrating mutation information from all mapped reads. The mutation ratio induced by mutagens was calculated as that remaining after subtraction of the average frequency of each mutation in control samples.

To facilitate detection of rare mutation events induced by the exposure to chemicals, we developed a novel approach to analyse the genome-wide likelihood of specific mutations within a heterogeneous bacterial cell population shortly after chemical exposure (Fig. 1). In our analyses, genomic DNA samples were directly extracted from heterogeneous bacterial cell populations without isolation of single cells, and read sequences were obtained. To minimise the sequencing error rate, only sequence information consistent between paired reads and within overlapping regions was included in the analysis. After mapping of read sequences, the ratios of each base substitution type were calculated separately for G:C or A:T base pairs by detecting all base substitution sites directly from mapped reads. Each base substitution type exhibited an error probability specific to its type (Supplementary Fig. S1); therefore, we calculated the increase in mutation frequencies per 10^6^ bp by subtracting the mutation ratios observed in control samples from those of mutagen-exposed samples. The resulting mutation frequencies per 10^6^ bp were considered to be representative of mutations induced by exposure to mutagen.

### Validation study using random DNA sequence samples

**Figure 2:**
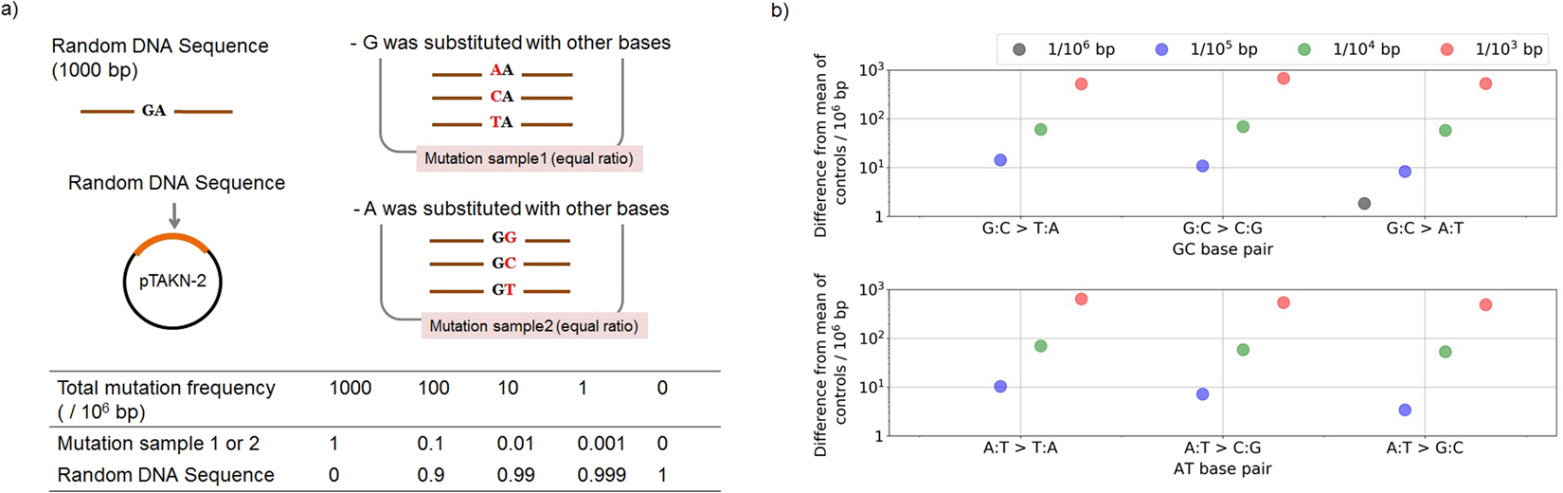
Validation experiment using random DNA sequences. (**a**) DNA samples with known numbers of mutations were created using a 1000-bp random DNA sequence. Briefly, the random sequence was synthesised, inserted into the pTAKN-2 vector, and used as the control sample. To mimic base substitutions, the same random DNA sequence, but with one of the G:C (501^st^) or A:T (502^nd^) base pairs replaced with another base pair, were also synthesised. These sequences were mixed at ratios of 0, 0.001, 0.01, and 0.1 to the control sample to create samples containing mutations with frequencies of 0, 1, 10, and 100 per 10^6^ G:C or A:T base pairs, respectively. As samples containing mutations with frequencies of 1000 per 10^6^ G:C or A:T base pairs, the replaced sequences themselves were used without addition to the control sample. Each sample contained the three types of base substitution of G:C or A:T base pairs in equal ratios. (**b**) Results of the analysis of mutation frequencies. The difference in mutation frequencies from the control sample is presented on a log scale.

To investigate the detection limit of our analytical flow, we first analysed DNA samples with known numbers of mutations, constructed using synthesised 1000 bp random DNA sequences (Fig. 2a, Supplementary Fig. S2). Each sample contained one G:C or A:T base pair substitution per 10^3^–10^6^ bp, with equal ratios of all three types of base substitutions of G:C or A:T base pairs (i.e., a transition and 2 transversions). The designed mutation frequencies for each mutation type corresponded to approximately 6.7 per 10^4^–10^7^ bp for each mutation type. After sequencing and base filtering, we obtained 1.45 $\pm$ 0.73 × 10^8^ bp of sequence data per sample, which corresponded to approximately 1.45 × 10^5^ depth per sample. The increases in mutation calls per 10^6^ bp were detected according to originally designed frequencies for both G:C and A:T base pairs (Fig. 2b). Differences in mutation frequencies from control samples were detected in all base substitution types included at frequencies of ≥1 per 10^5^ bp, whereas mutations in samples with frequencies of 1 per 10^6^ bp were only observed for the G:C > A:T transition; therefore, the detection limit of our method for each mutation type was determined as approximately 1 per 10^5^ bp. Among the six types of mutations, G:C > T:A, exhibited a relatively increased error frequency compared with other mutation types (Supplementary Fig. S1, S3).

### Analyses of mutations induced by alkylating agents

**Figure 3:**
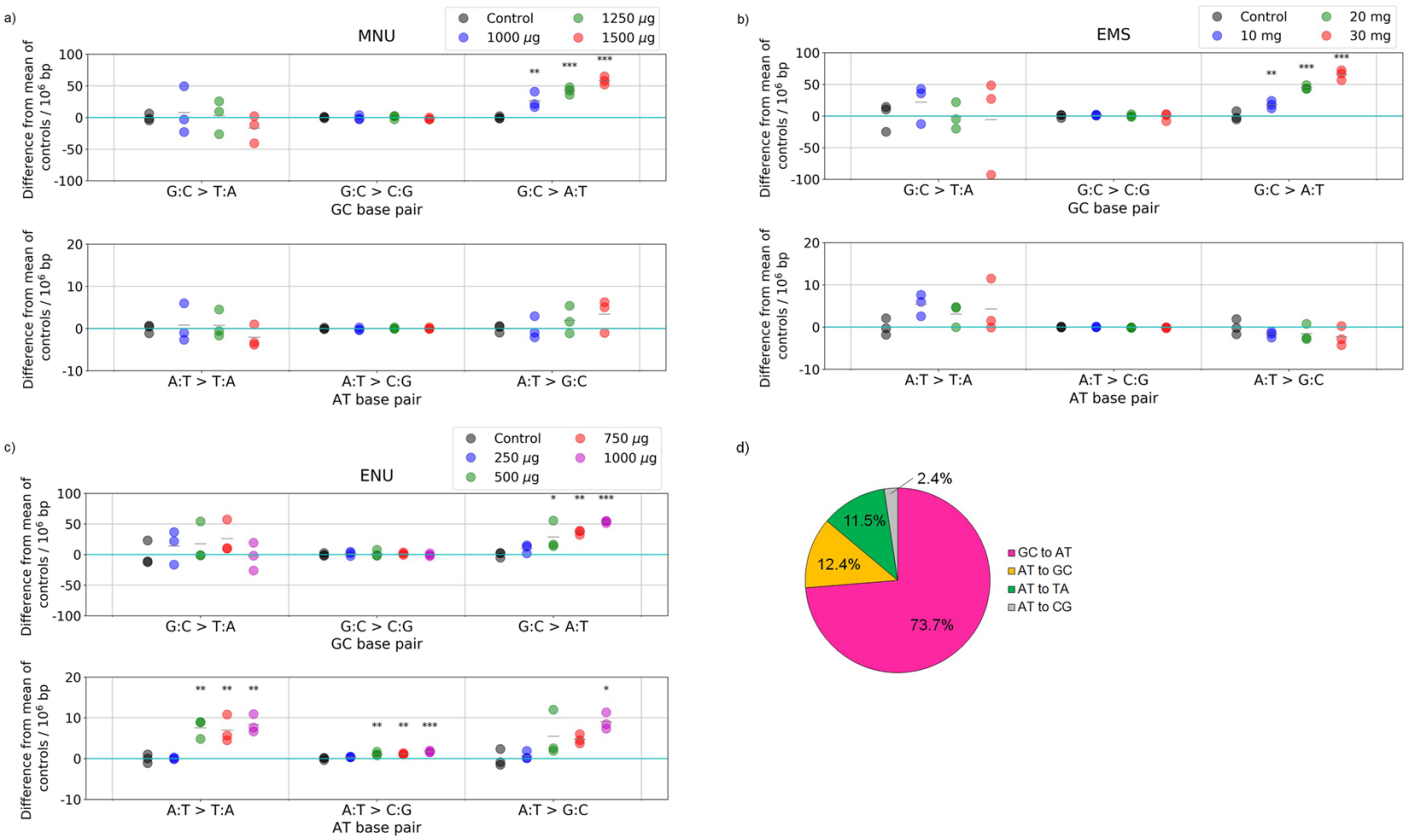
Analysis of the mutation frequencies induced by exposure to (**a**) MNU, (**b**) EMS, and (**c**) ENU. The differences in mutation frequencies per 10^6^ bp from that of the average of control samples is presented. Each circle represents the value of each sample within 3 independent biological experiments. A statistically significant increase in the frequency of G:C > A:T transition was observed in samples exposed to MNU, ENU, or EMS. In samples exposed to ENU, statistically significant increases in A:T > T:A and A:T > C:G transversions, and in the A:T > G:C transition, were also observed. Asterisks indicate p-values calculated using Dunnett’s multiple comparison test (*p < 0.05, **p < 0.01, and ***p < 0.001). (**d**) Mutation spectrum of samples exposed to ENU at the highest dose.

Genome DNA samples of *S*. *typhimurium* TA100 exposed to the alkylating agents MNU, EMS, and ENU were analysed using our analytical flow. These agents were adopted as representative mutagens because they are known to efficiently induce base substitutions. In addition, their mutational patterns are well-studied and they are known to exhibit different mutation patterns based on their mechanisms of action. The number of histidine-independent revertant colonies increased dramatically on exposure to alkylating agents (Table 1), indicating successful mutation induction. After genomic DNA sequencing and read filtering, we obtained 1.80 $\pm$ 0.15 × 10^7^, 2.88 $\pm$ 0.43 × 10^7^, and 3.06 $\pm$ 0.28 × 10^7^ of overlapping read pairs per sample for ENU-, MNU-, and EMS-exposed samples, respectively. These resulted in 5.57 $\pm$ 0.29 × 10^8^ bp, 5.72 $\pm$ 0.44 × 10^8^ bp, and 5.97 $\pm$ 0.28 × 10^8^ bp after base filtering, respectively. We first analysed the mutation pattern in the six-subtype format for comparison with those obtained by conventional short-term carcinogenicity assay. To characterise mutation patterns in the whole genome, we analysed differences in the frequencies of each type of base substitution in these DNA samples, based on the analytical flow described above. In the samples exposed to MNU or EMS, we detected statistically significant increases in G:C > A:T mutation frequencies (Fig. 3a–b). These results were consistent with the known mutation patterns observed in *S*. *typhimurium*^17,18^. In contrast, in samples exposed to ENU, we detected a significant increase in G:C > A:T mutation frequencies at G:C base pairs, whereas at A:T base pairs, we detected a significant dose-dependent increase in A:T > G:C and A:T > T:A mutations, along with a slight but significant increase in A:T > C:G mutations (Fig. 3c). The number of mutations was estimated to be a maximum of 70/10^6^ bp. Based on the increased frequencies of these mutations, we calculated the mutation spectrum induced by exposure to ENU at the highest dose (Fig. 3d). The results indicated that the most frequent mutation type was G:C > A:T, consistent with previous reports of analysis of ENU mutations in *Salmonella* strains^19,20^. We performed mutational analyses with a reduced amount of sequence data using sub-sampled sequence reads of ENU exposed samples. Statistically significant increase was detected at the same concentration as that in the original experiment in the samples using 1 M read pairs or more (i.e., 1 or 5 M read pairs), but not for samples using 0.3 M read pairs or less (i.e., 0.1 or 0.3 M read pairs). Thus, we found that our method minimally required approximately 1 million overlapping read pairs to achieve sufficient statistical power (Supplementary Fig. S4). This sequence amount was as small as that for the usual re-sequencing analysis of bacterial genome. Therefore, to our knowledge, our method most efficiently correctly identifies mutation spectra caused by exposure to alkylating agents.

**Table 1:** The results of Ames tests indicating the number of histidine-independent revertants of *S*. *typhimurium* TA100 induced by exposure to the alkylating agents, MNU, ENU, and EMS (n = 3).

Amount of test substance/tube		No. of colonies	
		Average	S.D.
DMSO		110	9.5
EMS (mg)	10	3345	308.9
	20	4886	302.2
MNU (μg)	750	3572	299.7
	1000	2936	29.3
ENU (μg)	500	3320	734.4
	1000	5051	172.9

### Sequence context analysis of mutations

**Figure 4:**
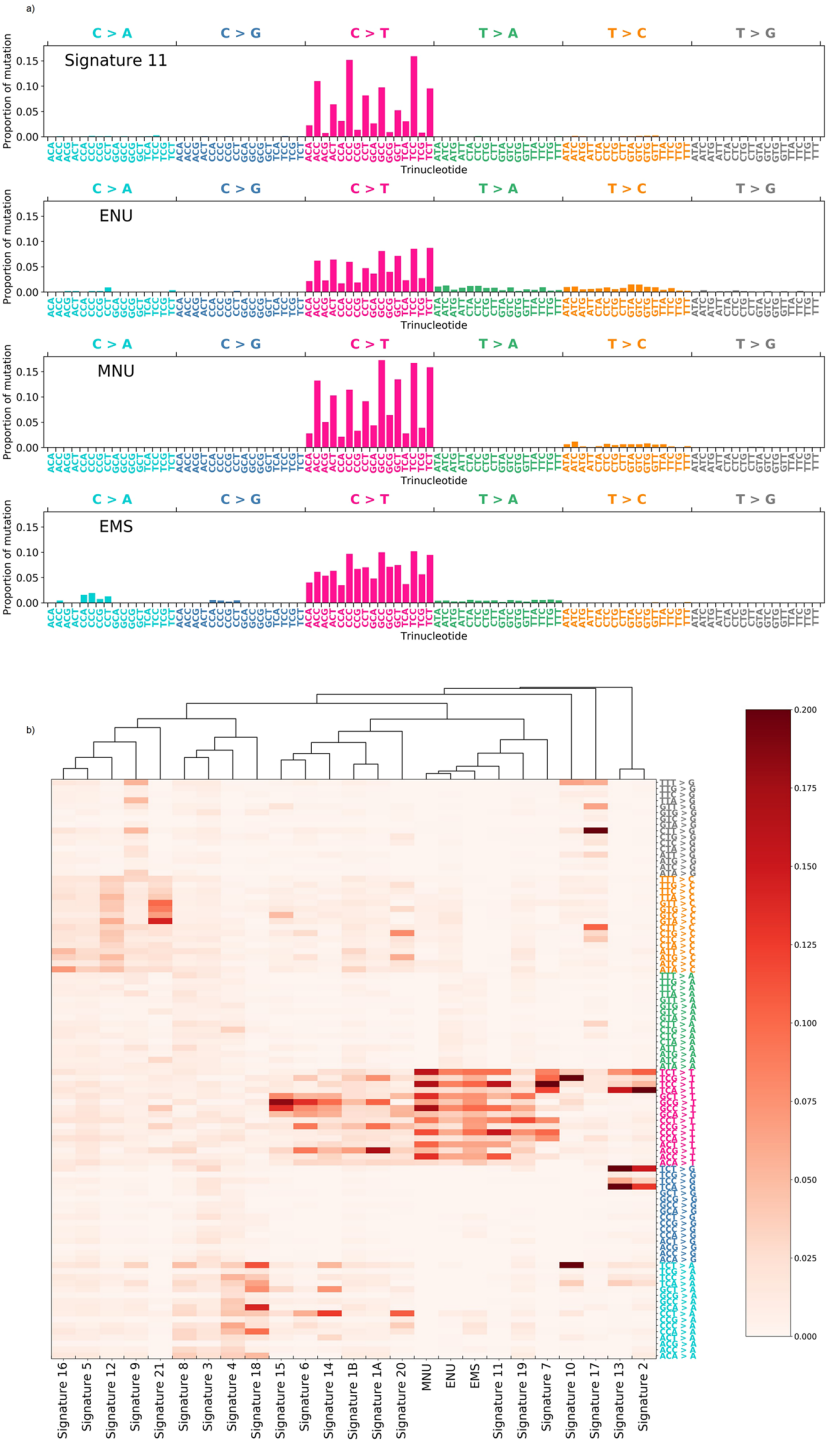
Results of sequence context analyses of mutations observed in samples exposed to alkylating agents. The average values calculated from the three independent experiments are shown. The frequencies of the six subtypes of base substitutions are shown in 96-trinucleotide format (**a**). All alkylating agents exhibited a peak in the context of the trinucleotide consensus, NpCpY (where Y indicates a pyrimidine base). The obtained mutation patterns were subjected to unsupervised hierarchical clustering (**b**). All patterns formed a sub-cluster with mutation signature 11 (alkylating agent) extracted from human cancers^6,21^.

Alexandrov *et al*. identified 21 distinct trinucleotide format mutational signatures from thousands of human cancer samples, and the signature induced by an alkylating agent (temozolomide) was identified as signature 11 in that report^6,21^. The mutational signatures of alkylating agents extracted from human cancers have not previously been confirmed by experiments using exposure to alkylating agents. We analysed the sequence context of mutations observed in samples exposed to the highest doses of MNU, EMS, or ENU, and analysed their similarity to the 21 distinct mutational signatures of human cancer. Figure 4 shows the ratio of increase in mutation frequencies of the six mutation subtypes in the context of each trinucleotide (each mutation type was represented by the pyrimidine bases; C and T). As a result, although these agents exhibited trinucleotide mutation patterns that differed slightly from one another, G:C > A:T mutations, which were the most frequently observed in these samples, tended to be more frequently observed in the context of the consensus sequence ‘NpCpY’ (where Y indicates a pyrimidine base). These patterns were similar to the trinucleotide mutation pattern of human cancer mutational signature 11^6,21^. Next, we performed hierarchical clustering of these patterns, together with the 21 human cancer mutational signatures validated by Alexandrov *et al*. The results indicate that all three patterns formed a cluster with signature 11, indicating that our method could identify the trinucleotide sequence changes characteristic of chemical exposure, as determined from signatures extracted from human cancer. Furthermore, these data suggest that our method provides a simple means to estimate the mechanism of action of each mutagen by obtaining sequence context data from observed mutations.

## Discussion

Our method emphasises the analysis of differences in mutation frequencies within the whole genome relative to control samples, rather than accurate identification of the position and type of each mutation. This alternative approach enables determination of precise mutation patterns within cell populations shortly after mutagen exposure, using a simple error mitigation technique.

The analytical sensitivity of our method was estimated as approximately 1 per 10^5^ bp for all types of base substitution. Our results from mutagen-exposed samples indicated that this was sufficiently high to permit analysis of mutations induced by well-understood mutagens in a bacterial strain. In our study, the background error frequencies of control samples were about 1 per 10^4–6^ bp depending on the mutation type, which is approximately the same as the mutation frequencies induced by mutagens; however, the variation in the background error frequency between samples and analyses was very small, enabling the detection of minor changes in mutation frequencies caused by chemical exposures (Supplementary Fig. S3), although a relatively high error frequency was observed for the G:C > T:A mutation call. We observed that this increase exhibited strand-specificity, occurring only when the read1 base was a G, rather than a C (Supplementary Fig. S3), indicating possible artificial modification of the G base. It has been reported that G is likely to be misread as a T nucleotide, due to the formation of 8-oxo-dG during NGS sample preparation^22,23^; therefore, it is possible that the sensitivity of G:C > T:A mutation detection was relatively strongly affected by this artificial error. The detection of mutations induced by mutagens that mainly cause G:C > T:A substitution, such as polycyclic aromatic hydrocarbons (PAHs), might be more difficult than detection of mutations caused by other types of mutagens^24,25^. Other library preparation and sequencing approaches which consider the sequence of the complementary strand, such as duplex sequences^15^, and circular consensus sequencing^26^, should improve sequence accuracy, although this would be at the expense of sequence efficiency. Adoption of these techniques should be considered based on a balance between the requirements for sensitivity and sequencing throughput for mutation evaluation.

Mutation analysis of samples treated with MNU, ENU, and EMS indicated that our method could correctly detect mutations generated by these agents in both a quantitative and qualitative manner. The number of mutations after ENU exposure has been reported as approximately 20–30 mutations per 10^6^ bp in an *S*. *typhimurium* strain^20^, which is the same order of magnitude as the total mutation frequency identified in our study. Qualitatively, alkylating agents are known to primarily cause G:C > A:T mutations; however, ENU can also specifically generate low levels of A:T base pair mutations in *S*. *typhimurium*^19^. In historical studies using Ames strains, although the number of detected mutations was limited, A:T > G:C was the most frequently reported A:T base pair mutation. In our study, an increase in A:T base pair mutations was observed in only samples exposed to ENU, validating the high resolution of our method. In addition, our results indicated approximately equal amounts of A:T > G:C and A:T > T:A mutations, which was not detected by historical analyses^17^. One reason for this discrepancy is likely the increased breadth of genomic target regions included in our mutation analysis. The mutation patterns obtained from a targeted single surrogate marker gene will have an increased chance of being influenced by limitations in sequence context diversity or the requirement for phenotypic change. In contrast, our method analysed mutation patterns on the whole-genome scale, providing a less biased representation of mutational patterns generated by mutagen exposures. Our method facilitates the generation of higher-quality mutation spectra of chemicals compared with those obtained by historical large-scale mutagenicity assays; therefore, the technique may be useful as a novel mutagenicity test, providing pure, high-resolution mutation patterns induced by chemical substances, which will be helpful in determining the mechanism of action for each mutagen.

The trinucleotide mutational pattern identified in our study recapitulated the mutational signature caused by temozolomide in human cancer^6,21^. Interestingly, the three alkylating agents exhibited similar, but slightly different, trinucleotide mutation patterns in our study. Several factors are considered to affect the mutation frequency in each trinucleotide context; for example, the reactivity of mutagens, the DNA repair machinery, and the effect of selection pressure during cancer development^27^. With the exception of certain mutagens, such as UV, little is known about the details of the mechanisms underlying the differences in mutation frequencies generated by various chemical mutagens in different sequence contexts. In our study, alkylating agents with the S_N_1 type reaction mechanism, i.e. MNU and ENU, exhibited a sharp peak for the NpCpY consensus sequence, similar to Signature 11 in human cancer (cosine similarities were 0.90 and 0.89, respectively). In contrast, EMS, which reacts with a mixed S_N_1/S_N_2 type mechanism, exhibited a broader peak relative to these agents (cosine similarity, 0.86)^28^. Because our method detects mutations after mutagen exposure without any selection process, the effect of selective pressure on this difference can be discounted; therefore, although further clarification is needed, these results suggest the involvement of different chemical mechanisms in generation of the various mutation frequencies in each trinucleotide context. Moreover, the data support the usefulness of analyses of the sequence context of mutated bases to facilitate high-resolution discrimination of mutagens based on their mechanisms of action. Because our method facilitates analysis of trinucleotide mutational patterns associated with specific mutagens, it has potential for use as a chemical mutagenicity test, providing quantitative and qualitative information about mutations. In this study, we identified some alkylating agents; however, analysing mutagens from other categories, such as nitroso compounds or aromatic amines, would provide a deeper understanding of the differences in the mutagenic mechanisms of different agents. Through analysis of the mutation patterns generated by a variety of mutagens using this method, we could extend systematic understanding of the mechanisms of action of mutagens, which has the potential to improve chemical safety assessments and regulations.

It is expected that additional mutational signatures will be extracted from numerous cancer genomes on a large scale in the near future. Such information would facilitate comprehensive understanding of the relationships between mutagens and cancer. Clarifying the biological processes underlying the extracted signatures will be the next challenge. To identify mutagens causing mutational signatures related to human cancer development, it is important to comprehensively understand the trinucleotide mutation patterns generated by various types of mutagen. The lack of a simple method to extract mutational signatures after mutagen exposure has impeded the progress of such research. Our results indicate that this technique could facilitate the identification of the causal mutagens underlying each mutational signature in human cancer. The mutation patterns obtained by our method might not coincide with mutation patterns in human cancer because DNA repair systems are not completely the same between bacteria and human cells. This possibility should be addressed by the application of our approach to human cells. By identifying signatures generated by a variety of mutagens using our method, it will be possible to connect each mutational signature identified in human cancer to causal mutagens. This would facilitate the understanding of the causes of specific cancers, and assist in the improvement of cancer prediction and prevention.

In conclusion, our novel approach to mutagenicity evaluation using NGS has potential as a high-throughput method for evaluation of trinucleotide mutation patterns generated by chemical exposures, and could reveal biological processes underlying human cancer by providing direct connections among mutations, mutagens, and cancer genomes. The characterisation of genomic mutations using both prospective and retrospective approaches could lead to early prediction of the causes of cancer and precise evaluation of the human carcinogenicity of specific chemicals.

## Materials and Methods

### Materials

Ethylnitrosourea (ENU; CASRN. 759-73-9) was purchased from Sigma-Aldrich (MO, U.S.). Methylnitrosourea (MNU; CASRN. 684-93-5) was purchased from Toronto Research Chemicals (Toronto, Canada). Ethyl methanesulphonate (EMS; CASRN. 62-50-0) was purchased from Tokyo Chemical Industry Co. Ltd. (Tokyo, Japan). Dimethyl sulphoxide (DMSO; CASRN. 67-68-5) was purchased from Wako (Osaka, Japan). The *Salmonella typhimurium* Ames tester strain, TA100, was obtained from the NITE Biological Resource Center (Tokyo, Japan).

### Construction of random DNA sequence samples

A 1000 bp random DNA sequence was synthesised and inserted into the pTAKN-2 vector by Eurofins Genomics (Tokyo, Japan) as a custom service. To mimic mutations, the same 1000 bp random DNA sequence but with one of the G:C (501^st^) or A:T (502^nd^) base pairs substituted with another base pair (i.e., T:A, C:G, and A:T for G:C, and T:A, C:G, and G:C for A:T) was also synthesised (Fig. 2, Supplementary Fig. S2). To create samples with known numbers of mutations, these mutation-carrying sequences were mixed in equal ratios and the resulting mixtures added to the original DNA sequence sample at ratios of 1, 0.1, 0.01, and 0.001, relative to the original DNA sequence. The total mutation frequencies in these samples corresponded to approximately 1 in 10^3^ to 1 in 10^6^ base pairs, respectively. The resulting materials were subjected to sequencing using the HiSeq 2500 platform.

### Ames test and bacterial DNA sample preparation

The TA100 strain was cultured for 4 h at 37 °C with Nutrient Broth No. 2 (Oxoid, UK) containing 24 µg/mL ampicillin (Sigma-Aldrich, MO, U.S.). The resulting bacterial cell suspensions were used for the Ames test and preparation of DNA samples exposed to alkylating agents (i.e., MNU, EMS, and ENU). Exposures to alkylating agents were performed according to the pre-incubation procedure of the standard Ames test^10^. Briefly, 100 µL of bacterial cell suspensions was mixed with 500 µL of phosphate buffer, pH 7.4 (Nacalai Tesque, Kyoto, Japan), and 100 µL of DMSO or test substance solution. The resulting mixture was incubated at 37 °C with shaking at 100 rpm for 20 min. After the incubation, to perform the Ames test, 2 mL of soft agar containing trace amounts of histidine and biotin was added to the mixture, and poured onto minimum glucose medium plates (Tesmedia AN; Oriental Yeast Co. Ltd., Tokyo, Japan). Plates were incubated at 37 °C for 48 h and the number of colonies generated was counted. To prepare DNA samples for sequencing, after chemical exposure, 50 µL of mixtures was inoculated into 2 mL of nutrient broth and cultured at 37 °C for 14 h to fix mutations. Subsequently, genomic DNA samples were isolated using a DNeasy Blood & Tissue Kit (Qiagen, Valencia, CA), according to the manufacturer’s instructions. Three independent experiments were performed for both Ames test and DNA sample preparations. Each DNA sample was subjected to sequencing analysis using the HiSeq 2500 platform.

### Sequencing of DNA samples and data processing

Library construction and sequencing by Illumina HiSeq were provided as a custom service of Eurofins Genomics K. K. (Tokyo, Japan). Briefly, random DNA sequences and genomic DNA samples were sheared to 150-bp fragments by sonication (Covaris, MA, U.S.). The resulting DNA fragments were processed for adaptor ligation and amplified to generate DNA libraries using a KAPA HTP Library Preparation Kit (Kapa Biosystems, MA, U.S.). Prepared libraries were sequenced on a HiSeq 2500 platform with v4 chemistry (Illumina, San Diego, U.S.). Adapter sequences and low quality bases were removed from the generated reads using Cutadapt^29^. Then, edited paired-end reads were merged with their overlapping regions using PEAR software^14^. Assembled reads were mapped to reference genome sequences and converted to SAM format using Bowtie2 software^30^. The pTAKN-2 vector sequence with the 1000 bp random sequence inserted was used as the reference genome sequence for the random DNA sequence samples. For TA100 genome samples, we used a TA100 strain-specific genome sequence obtained by re-sequencing the *S*. *typhimurium* LT-2 genome (GCA000006945.2) using GATK^31–33^. SAM format processing and pileup format conversion were performed using Samtools-1.2^34^. In the analyses using the *S*. *typhimurium* LT-2 genome, PCR duplicates were removed using Picard tools (http://broadinstitute.github.io/picard/, March 2018). Mapped bases were limited to those consistent in each read pair, and within overlapping regions of paired-end reads when converted to pileup format, based on their quality scores.

### Mutation detection and statistical analyses

To analyse mutation frequency, the number of each base substitution type was separately enumerated. The mutation rate for each mutation type per 10^6^ G:C or A:T bp was calculated by dividing each mutation count by the total read base count mapped to the G:C or A:T base pair, respectively. Statistical analyses were performed based on the frequencies of each mutation type per 10^6^ bp using Dunnett’s multiple comparison test. To estimate the minimum requirement for the sequencing amount to achieve sufficient statistical power, we sub-sampled 0.1, 0.3, 1, and 5 million overlapping read pairs from sequencing data of ENU-exposed samples and performed mutational analyses. To analyse the effects of mutagen exposure, the average values for control samples were subtracted from those of mutagen-exposed samples. To estimate the dependency of mutation frequencies on sequence context, the bases immediately 5′ and 3′ of each mutation were analysed and mutation rates were calculated in the context of each trinucleotide. The obtained 96 different trinucleotide format mutation patterns were subjected to hierarchical clustering analysis, along with the mutation patterns extracted from the human cancer genome reported by Alexandrov *et al*.^6^.

### Data availability

*S*. *typhimurium* strain sequence data used in this study are available at the DNA Data Bank of Japan Sequence Read Archive under Accession Number DRA006243.

## Electronic supplementary material

Supplementary figure
