# Supplementary material for: A genome-wide mutation analysis method enabling high-throughput identification of chemical mutagen signatures
Source: Sci Rep. 2018 Jun 25;8:9583. doi: 10.1038/s41598-018-27755-w (PMC6018237; doi:10.1038/s41598-018-27755-w)
Supplement: Supplementary file 1 — Supplementary figure [file 41598_2018_27755_MOESM1_ESM.pdf]

A genome-wide mutation analysis method enabling high-throughput identification of chemical mutagen signatures

Shoji Matsumura[1,*], Yurika Fujita[1], Masayuki Yamane[1], Osamu Morita[1], and Hiroshi Honda[1]

SUPPLEMENTARY DATA

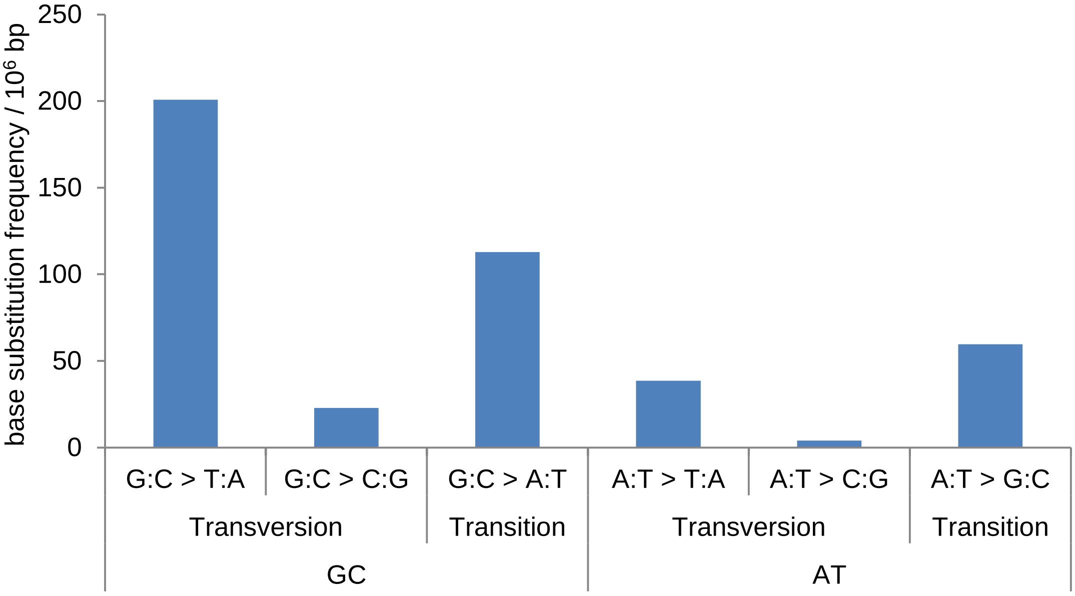

Figure S1

Base substitution frequencies per 10^6 bp for each base substitution type in control samples of a random DNA sequence. Each base substitution type exhibited a specific probability.

AAAATAGGACCGAGGAGTATGCATTCTTAGCGTTACCCTGGCGGGATTGTTGCCGA
AATAGGCTAATAGAACCAGAGATGTCAGGTACAACAGGGAGTTCGGCTCCCAGTCA
GGCTTGGCTCCCCACATGATGTGAACGTCTGAGCAAAGTAATAAAACATCGCGAGG
ATTGTCCCAACGTCCGACCGAGACGTGCATGGAGTTTGTGCGTCGATGCACTAATG
TTTAGGCTTCAACTACCAGAAAGACAACACTGTGTACCCCCTAAGCAAATGCTGAG
TATGCTTCACATGTTGAAAGAGTGCGTTAAATGTTAAGAAGAGCAAAAATCGCATG
CCGCGGTAGATATCATACTAACAAGTCGTGGCGATACAGGTGTACATTAGTCTAACC
GATGAACAAGGAAATTTAATCCGTCTACGGCCTTAGGTTCATCGTTTGTTCGGTGC
AGCTTTTCAATACAGAATGGTCGCTGCCTGCCATTATTTGTGAGTCGTTCT”GA”TG
CCTTTGTTTGTACGTATTTATCACGCTGTTAACGGTTTGCTGTCGTCGAAACTGCTA
CGATCTCGACGACCTCTGTTATCAGTATGGCCCTTGGCCACGGCAGCATATCTGTT
TTTAGGTTTAGTCGAGTGGCGCAACGAAAACTGCTCGGATTCTTGCCGACTAGCTG
GATCCAAAGCTAGCTTGTAGATACCTAGGTCCGTCCAGCGTACAAGCCTAGCGGTG
TCGAGCCTATACGCACTCACTGGGCTGTCCATGCATCCTGACCTTCTTGCACTTGTT
AATGTAGAAGGTGGCCAAGGGGAGGCCCACCCGCGGCTGACGGTCGTGACTATGG
GTGGGGGCGTGTAGCTGGCCACAAGTGAAGCGGCGGGAGCACTAATGGGCGCGT
GCCACAAAACTTAGCCGTAACCAAGTACGCTGTATAACTCACGTAAAAGGCCAGCG
CTTGGACATGGACCCGTGTCGGGATCACGCCGTAAAGCTATCCTTGCAA

Figure S2

The 1000 bp random DNA sequence used for validation studies. The 501st GC base pair or 502nd AT base pair was substituted for other bases for validation experiments.

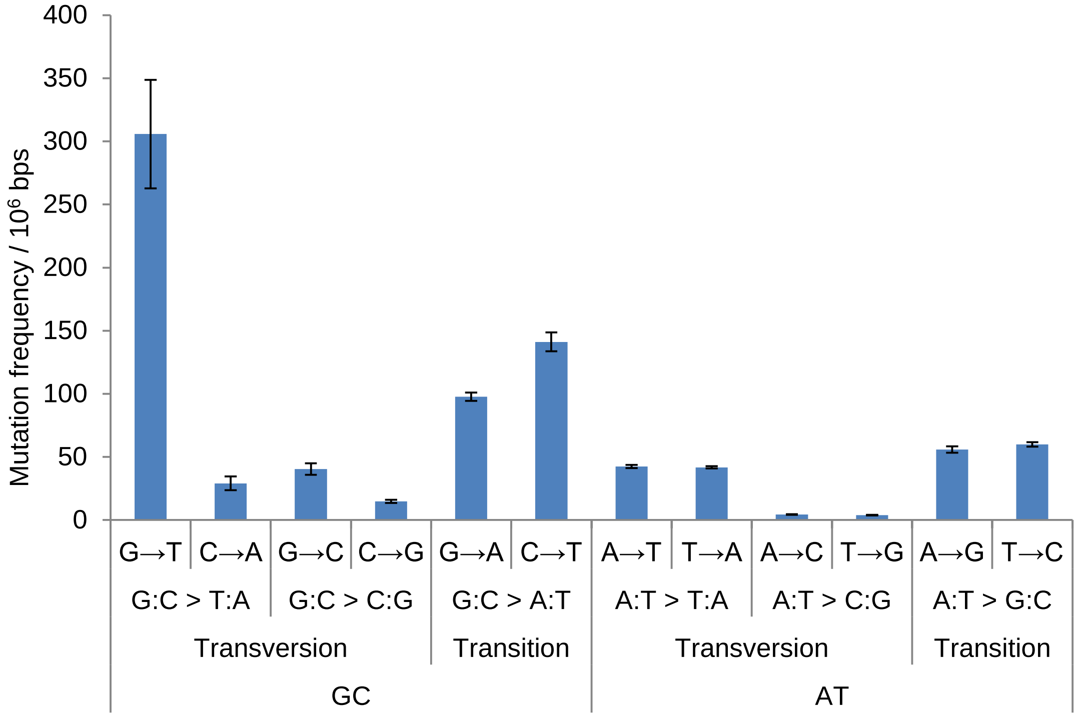

Figure S3

Base substitution frequencies per 10^6 bp for each type of base substitution in control TA100 DNA samples exposed to DMSO. The frequency of each base substitution was calculated separately, depending on the base to which the read1 sequence was mapped. The elevated frequency of G:C > T:A transversions exhibited strand specificity, in that it was more frequently observed when the read1 base was G.

a)

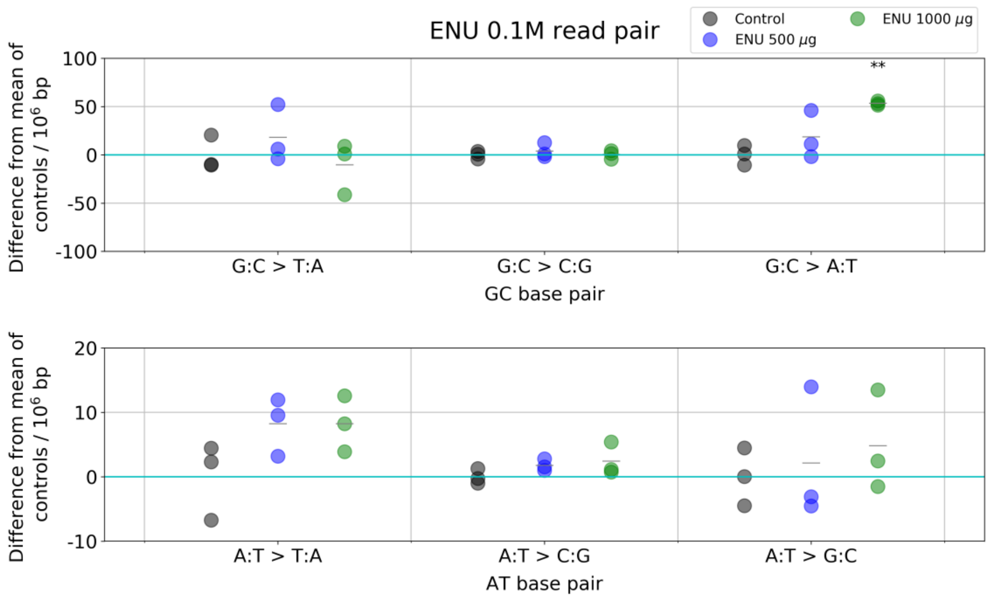
ENU 0.1M read pair
Control
ENU 500 μg
ENU 1000 μg
Difference from mean of controls / 10^6 bp
100
50
0
-50
-100
**
G:C > T:A
G:C > C:G
G:C > A:T
GC base pair
20
10
0
-10
A:T > T:A
A:T > C:G
A:T > G:C
AT base pair

b)

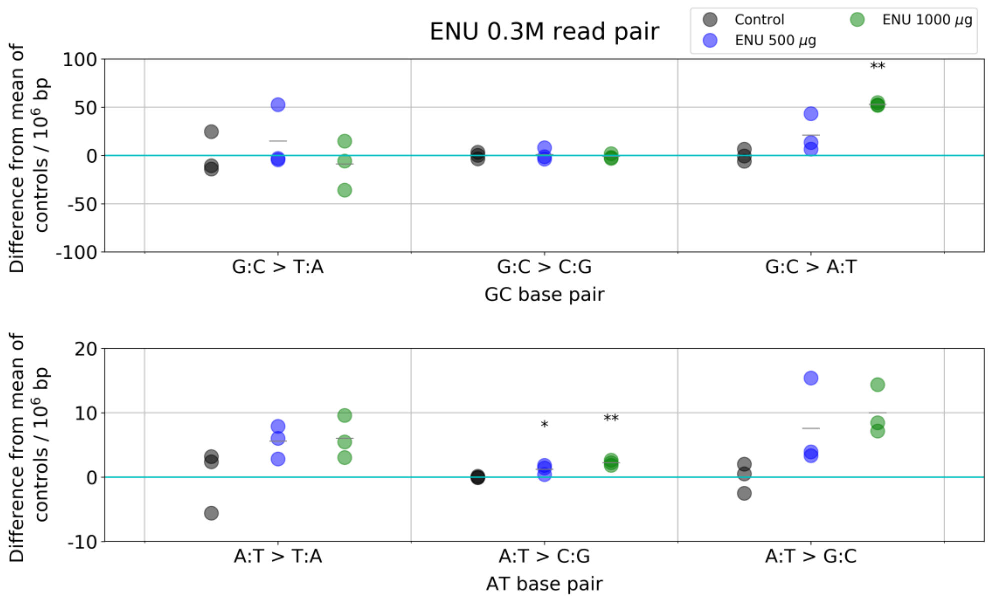
ENU 0.3M read pair
Control
ENU 500 μg
ENU 1000 μg
Difference from mean of controls / 10^6 bp
100
50
0
-50
-100
**
G:C > T:A
G:C > C:G
G:C > A:T
GC base pair
20
10
0
-10
*
**
A:T > T:A
A:T > C:G
A:T > G:C
AT base pair

c)

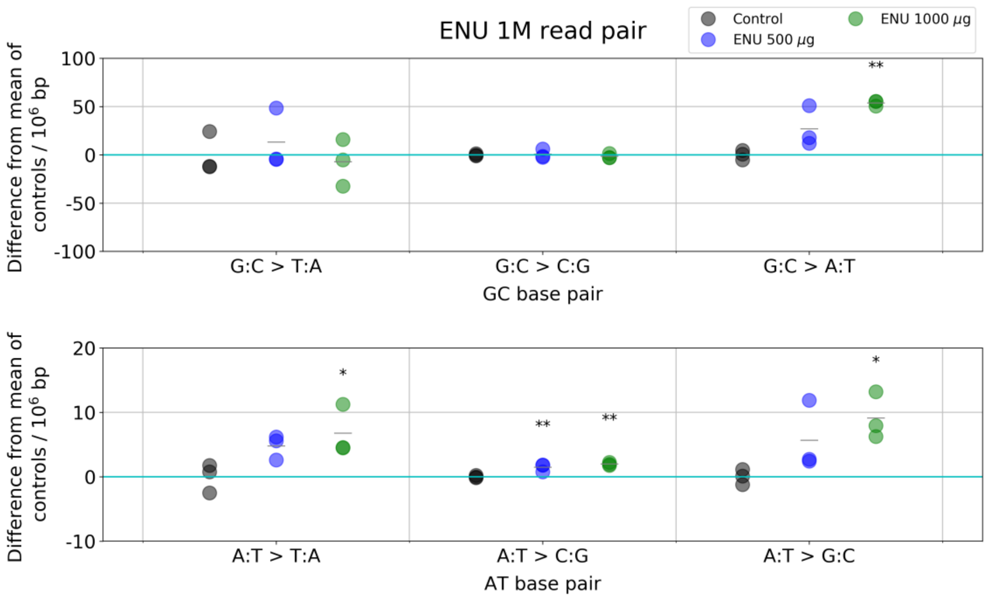

d)

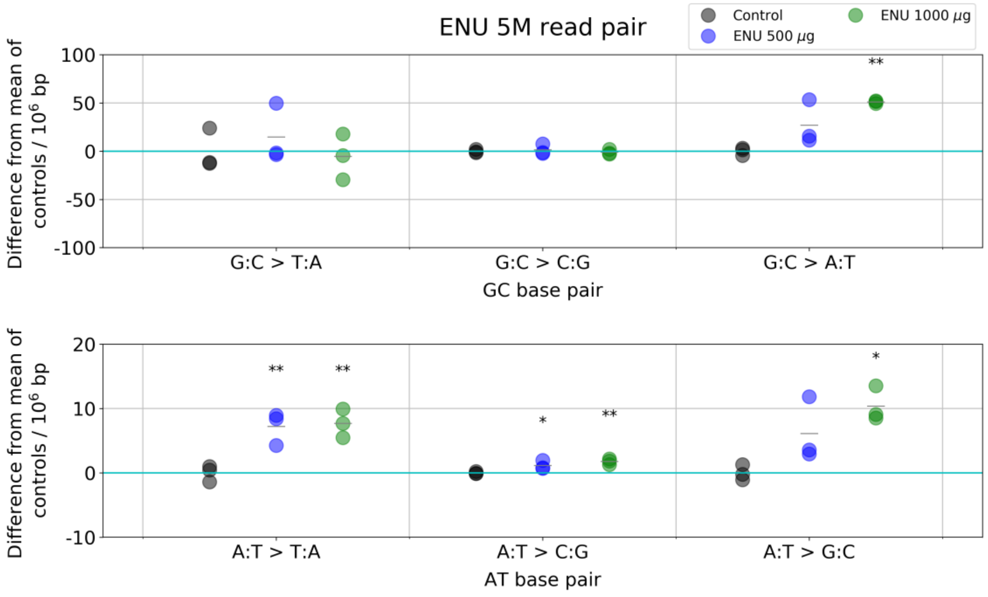

Figure S4

Analysis of the mutation frequencies induced by exposure to ENU using (a) 0.1, (b) 0.3, (c) 1, and (d) 5 M read pairs. The differences in mutation frequencies per 10^6 bp from the average value of control samples is presented. Each circle represents the value of each sample within 3 independent biological experiments. Statistically significant increase in

the frequency of G:C > A:T and A:T > G:C transition, and A:T > T:A and A:T > C:G transversions were consistently observed in the samples of 1 M or 5 M read pairs. Asterisks indicate p-values calculated using Dunnett's multiple comparison test (*$p < 0.05$, **$p < 0.01$, and ***$p < 0.001$).
